# MiR-200c downregulates HIF-1α and inhibits migration of lung cancer cells

**DOI:** 10.1186/s11658-019-0152-2

**Published:** 2019-04-27

**Authors:** Yuree Byun, Young-Chul Choi, Yunhui Jeong, Gangtae Lee, Sena Yoon, Yongsu Jeong, Jaeseung Yoon, Kwanghee Baek

**Affiliations:** Graduate School of Biotechnology, Kyung Hee University, Yongin, Republic of Korea

**Keywords:** microRNA, miR-200c, HIF-1α, Hypoxia

## Abstract

**Background:**

Hypoxia-inducible factor-1α (HIF-1α) is a transcription factor with a pivotal role in physiological and pathological responses to hypoxia. While HIF-1α is known to be involved in hypoxia-induced upregulation of microRNA (miRNA) expression, HIF-1α is also targeted by miRNAs. In this study, miRNAs targeting HIF-1α were identified and their effects on its expression and downstream target genes under hypoxic conditions were investigated. Cell migration under the same conditions was also assessed.

**Methods:**

microRNAs that target *HIF-1α* were screened using 3′-untranslated region luciferase (3′-UTR-luciferase) reporter assays. The expression levels of HIF-1α and its downstream target genes after transfection with miRNA were assessed using quantitative RT-PCR and western blot analyses. The effect of the miRNAs on the transcriptional activity of HIF-1α was determined using hypoxia-responsive element luciferase (HRE-luciferase) assays. Cell migration under hypoxia was examined using the wound-healing assay.

**Results:**

Several of the 19 screened miRNAs considerably decreased the luciferase activity. Transfection with miR-200c had substantial impact on the expression level and transcription activity of HIF-1α. The mRNA level of HIF-1α downstream genes decreased in response to miR-200c overexpression. MiR-200c inhibited cell migration in normoxia and, to a greater extent, in hypoxia. These effects were partly reversed by HIF-1α expression under hypoxic conditions.

**Conclusion:**

miR-200c negatively affects hypoxia-induced responses by downregulating HIF-1α, a key regulator of hypoxia. Therefore, overexpression of miR-200c might have therapeutic potential as an anticancer agent that inhibits tumor hypoxia.

**Electronic supplementary material:**

The online version of this article (10.1186/s11658-019-0152-2) contains supplementary material, which is available to authorized users.

## Background

Under hypoxic conditions, hypoxia-inducible factor-1α (HIF-1α) induces the transcription of multiple genes that are involved in processes such as angiogenesis, erythropoiesis, vascular tone, glucose metabolism, cell proliferation and apoptosis [1].

In normoxia, HIF-1α is hydroxylated on two proline (Pro) residues (Pro402 and Pro564) by a prolyl hydroxylase domain (PHD) protein [2, 3]. HIF-1α with hydroxyprolines is then recognized and bound by von Hippel-Lindau (VHL), an E3 ubiquitin ligase that ubiquitinates HIF-1α [4]. Subsequently, polyubiquitinated HIF-1α is degraded by the proteasome, resulting in the very low levels of HIF-1α protein observed at 21% oxygen.

In hypoxia, HIF-1α stabilizes and accumulates, dimerizing with HIF-1β and inducing the transcription of over 100 downstream genes. This leads to hypoxia-induced responses [5]. Since HIF-1α plays an important role in tumor development under hypoxic conditions by controlling angiogenesis, cell survival and metastasis, it is an attractive target for anti-cancer therapy [6].

It is well known that the miR-200 family (miR-200a, −200b, −200c, miR-429 and miR-141), plays an important role in epithelial–mesenchymal transition (EMT), which is important to tumor metastasis [7, 8]. Two transcriptional repressors of E-cadherin are among the direct targets of the miR-200 family: zinc finger E-box-binding homeobox 1 and 2 (ZEB1 and ZEB2). Since E-cadherin is a mediator of cell–cell adhesion and miR-200 upregulates E-cadherin by inhibiting ZEB1 and ZEB2, miR-200 can inhibit EMT.

Recent studies have reported that miR-200 plays a role in angiogenesis. It has been shown that V-ets erythroblastosis virus E26 oncogene homolog 1 (Ets-1), which promotes angiogenesis, is a direct target of miR-200b. The angiogenic activity of human microvascular endothelial cells is inhibited by miR-200b, as revealed using tube formation and wound-healing assays [9]. Another study, by Roybal et al., indicated that miR-200b downregulates murine *fms*-like tyrosine kinase (Flt-1 or VEGFR-1) by directly targeting its 3′-UTR, and consequently suppresses the invasion and metastasis of lung adenocarcinoma cells [10]. We previously reported that miR-200b downregulates three important players in angiogenesis in HUVECs: vascular endothelial growth factor (VEGF) and its receptors, Flt1 and kinase-insert domain-containing receptor (KDR or VEGFR-2) [11]. That study also showed that overexpression of miR-200b inhibits tube formation and VEGF signaling.

In this study, we show that HIF-1α, a key regulator of hypoxia signaling, is negatively regulated by miR-200c in lung carcinoma cells. Since HIF-1α induces the transcription of over 100 hypoxia-responsive genes at low oxygen levels and tumor hypoxia is important for tumor growth and metastasis, miR-200c may provide an attractive approach for potential anti-cancer therapies through the inhibition of multiple hypoxia-induced signaling pathways, including angiogenesis.

## Materials and methods

### RNA oligonucleotides and transfection

**Fig. 1:**
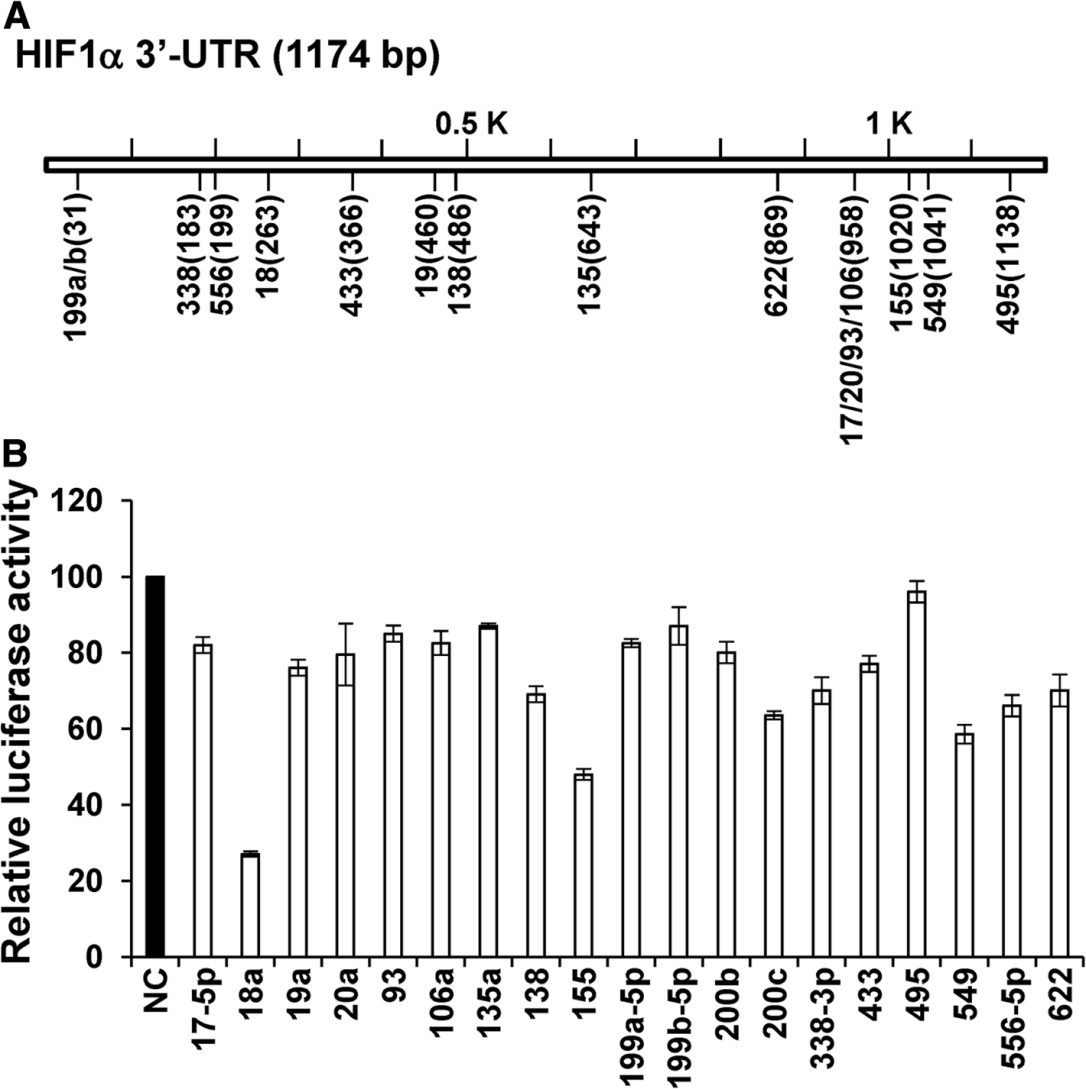
Screening for miRNAs that target the 3′-UTR of *HIF-1α.* **a** – Schematic depiction of the TargetScan predictions for the binding sites for miRNAs in the 3′-UTR of *HIF-1α*. The locations of miRNA-binding sites in the 3′-UTR of the *HIF-1α* gene are represented by numbers in parentheses. **b** – Luciferase reporter assay. A549 cells were co-transfected in duplicate with 3′-UTR-luciferase reporter plasmid and miRNA mimics. The luciferase activity was measured 48 h post-transfection. Luciferase activity in NC-transfected cells was set at 100%

miRNA mimics, short interfering RNAs (siRNAs) and a negative control (NC) miRNA were purchased from Shanghai GenePharma Company. Cells were transfected with miRNA mimics and siRNAs (20 nM) using Lipofectamine RNAiMAX (Invitrogen), following the manufacturer’s instructions. The 19-mer target sequences of the siRNAs targeting HIF-1α were: siR783–5′-CTAACTGGACACAGTGTGT-3′; and siR2210–5′-CCAGCAGACTCAAATACAA-3′. For screening experiments (Fig. 1a), 17 miRNAs were selected based on evolutionary conservation using the target prediction software TargetScan: miR-17-5p, −18a, −19a, −20a, − 93, −106a, −135a, − 138, − 155, −199a-5p, −199b-5p and − 338-3p are conserved among vertebrates; miR-433 and -495 are conserved only among mammals; and miR-549, − 556-5p, and − 622 are poorly conserved). MiRNA-200b/c, which are not predicted to target HIF-1α, were used as the control miRNAs.

### Cell culture

The human cell lines A549 (lung carcinoma), NCI-H460 (lung carcinoma) and MCF-7 (breast carcinoma) were obtained from the Korean Cell Line Bank. The cells were cultured in RPMI-1640 supplemented with 10% fetal bovine serum, 100 U/ml penicillin, and 100 μg/ml streptomycin and incubated at 37 °C in a humidified incubator containing 5% CO_2_.

To chemically induce HIF-1α, the cells were treated with 200 μM of the HIF-1α-stabilizing compound cobalt chloride (CoCl_2_) for 24 h at 21% oxygen. Hypoxic conditions were simulated in a hypoxia chamber (MIC-101; Billups-Rothenberg) containing 1% O_2_, 5% CO_2_, and 94% N_2_ at 37 °C. For hypoxic experiments, cells were treated with CoCl_2_ or incubated in a hypoxic chamber 24 h post-transfection. After 24 h in hypoxia, cells were harvested for quantitative RT-PCR and western blot analyses.

### Western blot analysis

Western blotting was performed as described previously [12]. Primary antibodies specific for HIF-1α (mouse monoclonal; 610958) and β-actin (goat polyclonal; C-11) were purchased from BD Biosciences and Santa Cruz Biotechnology, respectively.

### Construction of 3′-UTR reporter plasmids and luciferase assays

The 3′-UTR of *HIF-1α* (NM_001530) was amplified from the full-length cDNA obtained from Open Biosystems via PCR using the following primers: HIF-1α-F, 5′-GAT CTC GAG GCT TTT TCT TAA TTT CAT TCC T-3′ and HIF-1α-R, 5′-GAT GCG GCC GCG CCT GGT CCA CAG AAG ATG TTT A-3′. After digestion with XhoI and NotI, the 3′-UTR fragment was cloned into the XhoI/NotI sites of the psiCHECK-2 vector (Promega) to obtain a 3′-UTR-luciferase reporter plasmid. To eliminate the predicted miR-18 and miR-549 target sites from the reporter plasmid, PCR was applied as previously described [13], using the following primers: HIF-1α-F/HIF-18-R and HIF-18-F/HIF-1α-R for miR-18; and HIF-1α-F/HIF-549-R and HIF-549-F/HIF-1α-R for miR-549. The primer sequences were: HIF-18-R; 5′-GATAAGCTTATTTTTTAAAATGATGCTAC-3′, HIF-18-F; 5′-GATAAGCTTTATTTATTTATTTTTGGCTA-3′, HIF-549-R; 5′-GATGAATTCATATATTCCTAAAATAATGCTT-3′, HIF-549-F; 5′-GATGAATTCCAGTAAATATCTTGTTTTTTCTA-3′. The DNA fragments amplified using the described primer pairs were digested with HindIII (miR-18) and EcoRI (miR-549). The digested fragments were then ligated at 4 °C overnight, digested with XhoI and NotI, and cloned into the psiCHECK-2 vector.

Luciferase assays were performed via cotransfection with 250 ng of 3′-UTR-luciferase reporter plasmid and miRNA mimics (10 nM) using Lipofectamine 2000 (Invitrogen). The A549 cells were assayed 48 h post-transfection for firefly and Renilla luciferase activities using the dual-luciferase assay (Promega). The Renilla luciferase values were then divided by the firefly luciferase activity values to normalize the difference in transfection efficiency. The experiments were performed in triplicate and repeated three times.

### HRE-luciferase reporter assays

The hypoxia-responsive element luciferase (HRE-luciferase) reporter plasmid containing three HREs (24-mers) from the phosphoglycerate kinase 1 (PGK1) gene (#26731) was obtained from Addgene. For luciferase assays, A549 cells were seeded at a density of 7 × 10^4^ cells/well in 12-well plates. The following day, cells were co-transfected with 120 ng HRE-luciferase reporter plasmid, 20 ng pGL4.75 plasmid (Promega), and 20 nM miRNA. Firefly and Renilla luciferase activities were assayed 48 h post-transfection using a dual-luciferase assay kit (Promega). The Renilla luciferase activity produced from the pGL4.75 plasmid was used for normalization. The experiments were performed in triplicate and repeated three times.

### Quantitative PCR analysis

Total RNA was isolated using the RNeasy Mini kit (Qiagen). We used 1 μg of total RNA to synthesize cDNA using the iScript cDNA synthesis Kit (Bio-Rad). Expression levels were determined using quantitative RT-PCR, which was performed twice in triplicate in 384-well plates using the ABI Prism 7900 Sequence Detection System (Applied Biosystems). Reaction mixtures contained 10 μl of 2× SYBR Green PCR Master Mix (Applied Biosystems), 0.8 μl of primer mix (10 pmol/μl), and 1 μl cDNA. Thermal cycling conditions were as follows: 95 °C for 10 min, followed by 40 cycles of 95 °C for 30 s, 60 °C for 30 s, and 72 °C for 30 s. The expression of each cDNA was normalized to that of actin, and data analysis was performed with the comparative Ct method [14]. The primers used for quantitative RT-PCR are shown in Additional file 1: Table S1.

### DNA methylation analysis

Genomic DNA was isolated from cultured cells using the Wizard Genomic DNA purification kit (Promega). A bisulfite treatment was performed with 0.7 μg of genomic DNA using the EZ DNA Methylation-Gold kit (Zymo Research). After purification, PCR was carried out using 0.2 μg of bisulfite-converted genomic DNA. Each reaction contained 8 pmol each of primers and the EpiMark Hot Start Taq DNA Polymerase (New England BioLabs) in a 25 μl reaction volume. The amplification cycle consisted of initial denaturation at 95 °C for 60 s, followed by 40 cycles of 95 °C for 25 s, 55 °C for 45 s, and 68 °C for 45 s. The sequences of primers were: 200c forward primer; 5′-TAGGTAAAGGTTATTAGGGGAGAGG-3′ and 200c reverse primer; 5′- AACCCAAATTACAATCCAAACAA-3′.

These primers helped to amplify the CpG island sequence that is located approximately 300 bp upstream of miR-200c, as predicted by the MethPrimer software (Fig. 3a). PCR products were purified using the DNA Clean and Concentrator kit (Zymo Research) and cloned into the pGEM-T easy vector (Promega). Between 10 and 20 recombinant clones per sample were sequenced.

### Quantitative RT-PCR analysis of miRNA

Total RNA, including miRNAs, was isolated using the mirVana miRNA isolation kit (Ambion, Thermo Fisher Scientific). From 2 μg of total RNA, first strand cDNA was synthesized using the miScript II RT Kit (Qiagen). For PCR, primer pairs (miR-200c-3p and RNU6–2) were obtained from Qiagen and quantitative RT-PCR was performed twice in triplicate on the StepOnePlus Real Time PCR System (Applied Biosystems) using the 2X QuantiTect SYBR Green PCR Master Mix (Qiagen), according to the manufacturer’s instructions. Thermal cycling conditions were: 95 °C for 15 min, followed by 40 cycles of 94 °C for 15 s, 55 °C for 30 s, and 70 °C for 30 s. The data were analyzed using the StepOne software v2.2.2 (Applied Biosystems). The expression level of miR-200c was normalized to that of U6 small nuclear RNA and calculated using the 2^-ΔΔCt^ method.

### Transfection of miR-200c inhibitor

MiR-200c inhibitor and a negative control inhibitor (NC inhibitor) were purchased from Shanghai GenePharma Company. MCF-7 cells were transfected with miR-200c inhibitor and NC inhibitor (100 nM). The next day, cells were incubated under normoxic or hypoxic conditions. After a 24-h culture, cells were harvested, total protein was isolated, and the levels of HIF-1α were determined via western blot analysis.

### Wound-healing assay

A549 cells were transfected with miR-200c mimic and siR783 (20 nM) and grown to 100% confluence. A scratch was applied to transfected cells using a 200-μl pipette tip and the cells were incubated for 43 h in complete culture medium. Images were taken at 0 and 43 h after wounding, and the distance between the wound edges was measured using the Leica Application Suite version 3.8.0 on images captured with the Leica microscope. The experiments were performed in triplicate and repeated three times.

For rescue experiments, the open reading frame (ORF) of HIF-1α was amplified using PCR from the full-length cDNA (Open Biosystems) using the primers HIF-1α-ORF-F (5′-GATGGATCCATGGAGGGCGCCGGCGGCGC-3′) and HIF-1α-ORF-R (5′-GATTCTAGATCAGTTAACTTGATCCAAAGCTC-3′). After digestion with BamHI and XbaI, the ORF fragment was cloned into the pcDNA3.1 vector to obtain the pcDNA3.1-HIF-1α plasmid. Transfection was performed as previously described [12] with miR-200c (20 nM) and pcDNA3.1 or pcDNA3.1-HIF-1α plasmid (25 ng/12 well) using Lipofectamine RNAiMAX and TransIT-X2 (Mirus Bio).

### Statistical analysis

Data are shown as the means ± standard deviation (SD). Differences between groups were analyzed using the two-tailed Student’s t-test. *p* < 0.05 was considered statistically significant.

## Results

### Screening for miRNAs targeting HIF-1α using 3′-UTR-luciferase reporter assay

**Fig. 2:**
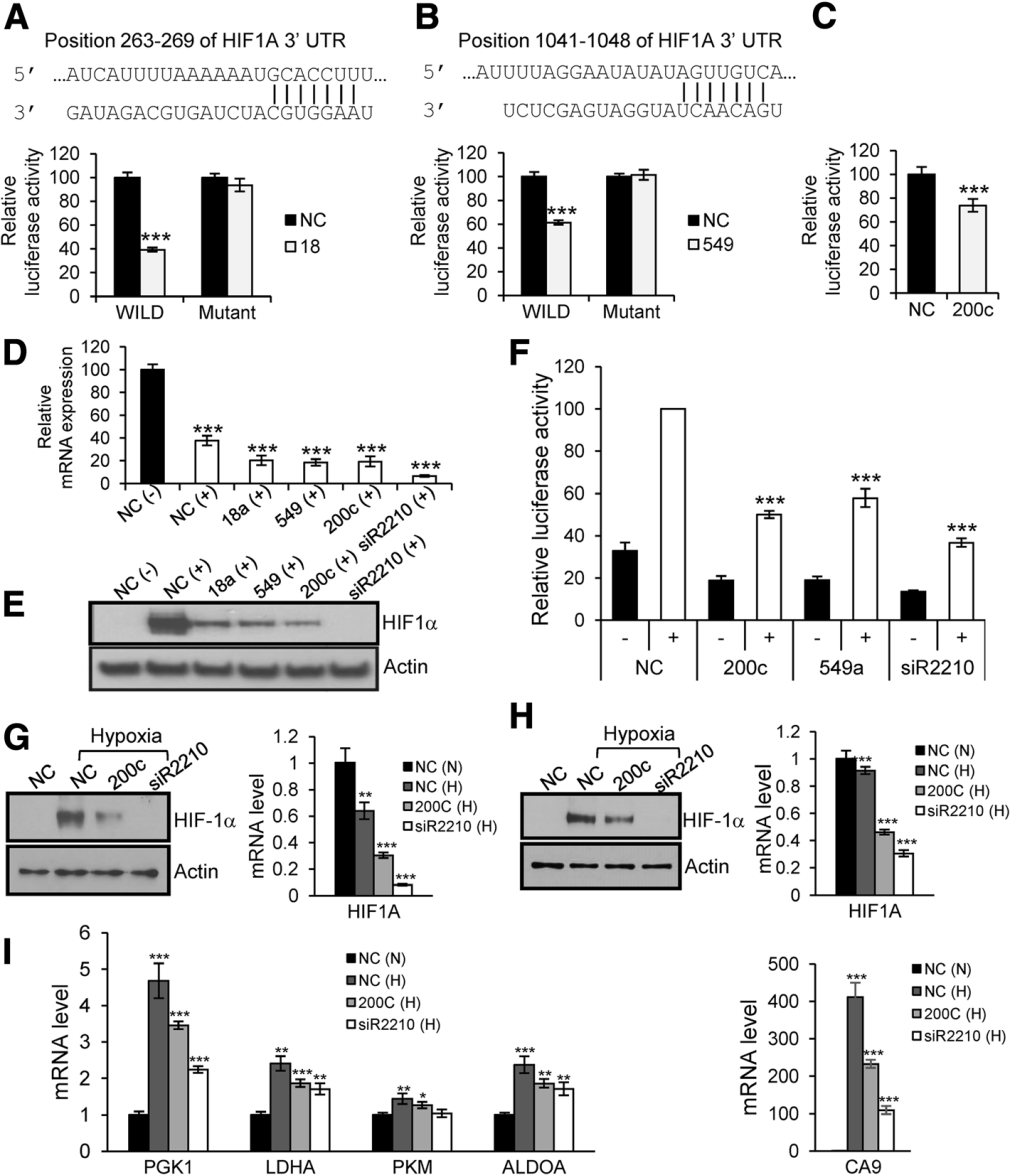
Downregulation of HIF-1α by miR-18, −200c and − 549. **a** and **b** – miR-18 (**a**) and miR-549 (**b**) downregulate HIF-1α by targeting predicted binding sites in the 3′-UTR of *HIF-1α.* Luciferase activity in NC-transfected cells was set at 100%. ***p < 0.001. **c –** Luciferase activity decreased following co-transfection with miR-200c and wild-type 3′-UTR-luciferase plasmid. **d** – A549 cells were transfected with miRNA mimics and siRNA against *HIF-1α* (20 nM). The next day, cells were treated with CoCl_2_ (200 μM). After 24 h, total RNA was isolated and subjected to quantitative RT-PCR. (−): no treatment with CoCl_2_; (+): treatment with CoCl_2_. ***p < 0.001. **e** – A549 cells were transfected and treated with CoCl_2_ as indicated in (**d**). After treatment with CoCl_2_, western blot was performed using total cell lysate. **f** – The transcriptional activity of HIF-1α was measured after co-transfection of cells with the HRE-luciferase plasmid, pGL4.75 plasmid and miRNA mimics. The luciferase activity was assayed 48 h after transfection. -, no treatment with CoCl_2_; +, treatment with CoCl_2_. ***p < 0.001. **g** and **h** – Downregulation of HIF-1α by miR-200c in hypoxia. The mRNA and protein levels of HIF-1α are downregulated by miR-200c in A549 (**g**) and NCI-H460 (**h**) lung carcinoma cells under hypoxic condition. N; normoxia, H; hypoxia. siR2210 was used to silence HIF-1α in the western blot and quantitative RT-PCR analyses. In quantitative RT-PCR, the mRNA level of HIF-1α obtained from NC-transfected cells in normoxia was set as 1. **p < 0.01, ***p < 0.001. **i** – Downregulation of downstream target genes by miR-200c in hypoxia. The mRNA level of downstream target genes in A549 cells was assessed using quantitative RT-PCR after transfection with miR-200c and siR2210. N; normoxia, H; hypoxia. The mRNA level of target genes from NC-transfected cells in normoxia was set as 1. *p < 0.05, **p < 0.01, ***p < 0.001

To screen for miRNAs targeting *HIF-1α*, A549 cells were co-transfected with each mature miRNA mimic and a luciferase reporter plasmid containing the 3′-UTR of *HIF-1α*. Of the 19 miRNAs identified, miR-18, miR-155, and miR-549 significantly downregulated luciferase activity (Fig. 1). This decrease in luciferase activity was abrogated after we mutated a predicted target site for miR-18 and miR-549 in the 3′-UTR of *HIF-1α*, indicating that miR-18 and miR-549 downregulate HIF-1α by binding to the predicted target sites in the 3′-UTR of *HIF-1α* (Fig. 2a and b)*.* Unexpectedly, cotransfection with miR-200c, which does not have a target sequence in the 3′-UTR of *HIF-1α,* resulted in substantial downregulation of luciferase activity (Fig. 2c). Since the finding that miR-200c regulates HIF-1α expression is an extension of our previous study reporting that miR-200b/c negatively affects angiogenesis by targeting VEGF and its receptors [11], we focused on miR-200c for further investigations, together with miR-18 and miR-549, which served as positive controls.

### MiR-18, − 549 and -200c downregulate the mRNA and protein levels of HIF-1α

To verify if HIF-1α is a target of miR-18, − 549 and -200c, we examined the downregulation of HIF-1α by the three miRNAs using quantitative RT-PCR and western blot analyses. Since the protein level of HIF-1α is very low in normoxia, we used CoCl_2_, which stabilizes HIF-1α by preventing its ubiquitination and proteasomal degradation [15].

The RNA levels of *HIF-1α* decreased in NC-transfected cells treated with CoCl_2_ (Fig. 2d) and further decreased in CoCl_2_-treated cells transfected with miR-18, − 549 or -200c. Silencing of *HIF-1α* after transfection with siR2210, which targets *HIF-1α,* was associated with very low levels of *HIF-1α* mRNA, indicating high knockdown efficiency. When protein levels were examined using western blot analyses (Fig. 2e), we found that HIF-1α stabilized and accumulated in NC-transfected cells treated with CoCl_2_, while its levels decreased in cells transfected with miR-18, − 549 or -200c and treated with CoCl_2_. Additionally, HIF-1α was not detected in cells transfected with siR2210 targeting *HIF-1α.* These results indicated that miR-18, − 549 and -200c downregulate HIF-1α at the mRNA and protein levels.

### MiR-549 and -200c downregulate the transcriptional activity of HIF-1α

To examine whether miR-549 and -200c negatively affect the transcriptional activity of HIF-1α, A549 cells were co-transfected with the HRE-luciferase reporter plasmid, the pGL4.75 plasmid and miRNA mimics. The activity of firefly and Renilla luciferase was determined 48 h post-transfection. As targeting of *HIF-1α* by miR-18 was previously reported in other cell types [16, 17], miR-18 was excluded from these experiments. In NC-transfected cells, treatment with CoCl_2_ resulted in an increase in luciferase activity, indicating induction of the transcriptional activity of HIF-1α (Fig. 2f). In cells transfected with miR-200c or miR-549, the luciferase activity decreased compared to the NC-transfected cells. Transfection with siR2210 (the control) resulted in even lower luciferase activity. These results indicate that the transcriptional activity of HIF-1α is downregulated by miR-200c and miR-549.

### Downregulation of HIF-1α and its downstream target genes by miR-200c in hypoxia

Consistent with the results observed in CoCl_2_-treated cells, the levels of HIF-1α substantially decreased in A549 and NCI-H460 cells after transfection with miR-200c under hypoxic conditions (Fig. 2g and h).

To examine whether HIF-1α downstream target genes are downregulated by overexpression of miR-200c, we performed additional quantitative RT-PCR assays. Specifically, we investigated the expression of genes involved in glucose metabolism: PGK1, lactate dehydrogenase A (LDHA), pyruvate kinase muscle (PKM), and fructose-bisphosphate aldolase (ALDOA); and one gene involved in the hydration of CO_2_: carbonic anhydrase 9 (CA9). As shown in Fig. 2i, in NC-transfected A549 cells, the mRNA level of these genes increased in hypoxia (compare “N” with “H”). When the cells were transfected with miR-200c or siR2210 under hypoxic conditions, the expression level of HIF-1α target genes decreased. In the case of CA9, which is a known marker of hypoxia and is induced by HIF-1α [18, 19], the mRNA levels were greatly upregulated in hypoxia and decreased upon transfection of the cells with miR-200c or siR2210. These results indicated that miR-200c adversely affects the hypoxia-induced response by downregulating HIF-1α and its downstream target genes.

### DNA methylation patterns and expression of miR-200c in several types of cells

**Fig. 3:**
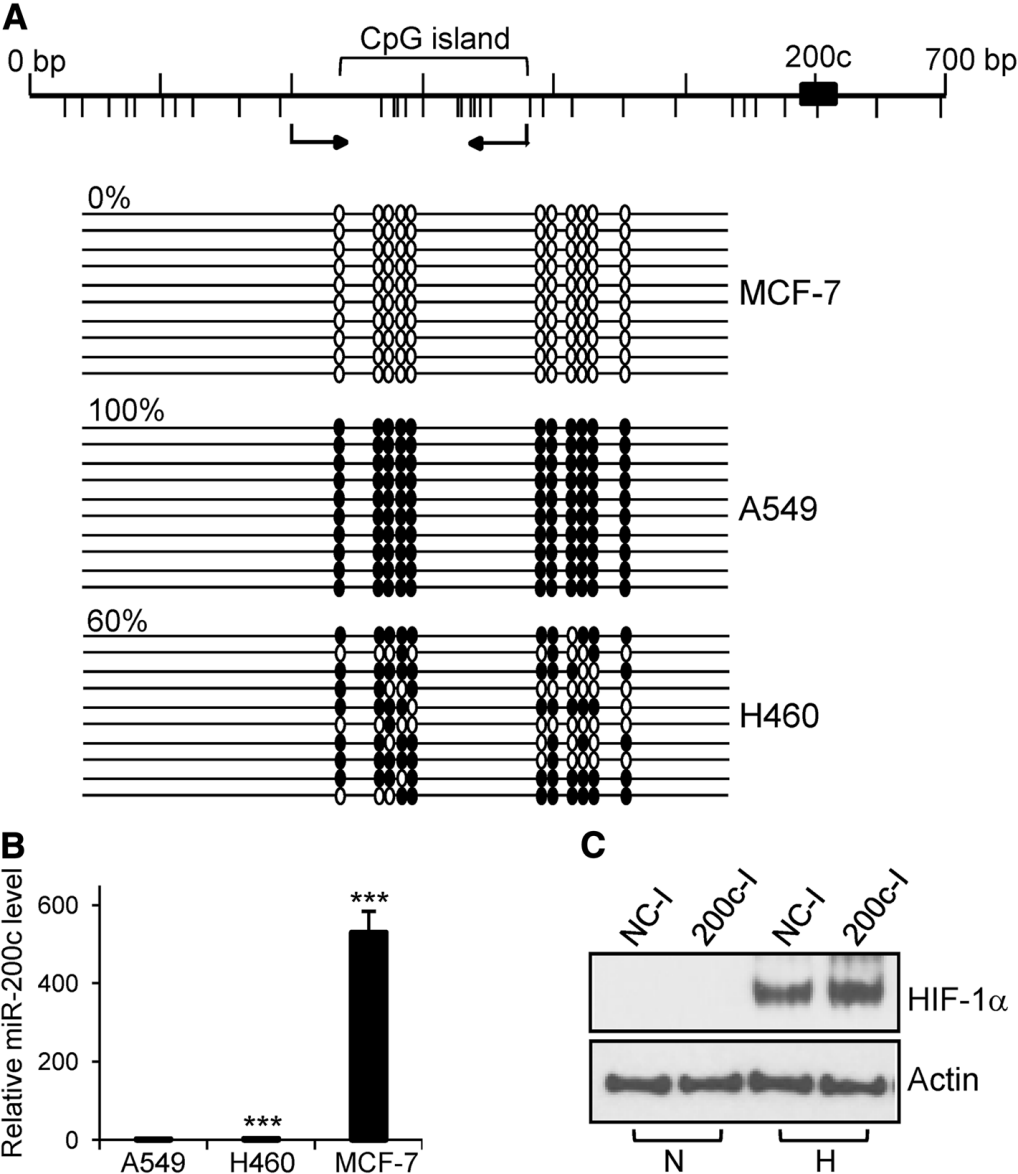
DNA methylation and the expression of miR-200c. **a** – The DNA methylation pattern of a CpG island located upstream of miR-200c was examined using bisulfite sequencing. CpG dinucleotides are depicted using vertical bars below the map. The DNA region analyzed via bisulfite sequencing is indicated by the arrows below the map. The methylated and non-methylated CpG sites are indicated by filled and open circles, respectively. Each line represents an independent clone. Percentages indicate the level of DNA methylation. **b** – The expression of miR-200c was assessed via quantitative RT-PCR. The expression level of miR-200c in A549 cells was set at 1. ***p < 0.001. **c** – Upregulation of HIF-1α protein after transfection with a miR-200c inhibitor. MCF-7 cells were transfected with NC inhibitor or miR-200c inhibitor. The following day, the transfected cells were incubated in normoxia and hypoxia. After 24 h, total proteins were isolated and subjected to western blot analysis. N; normoxia, H; hypoxia, NC-I; NC inhibitor, 200c-I; miR-200c inhibitor

Since DNA methylation inversely correlates with gene expression [20, 21], we examined the DNA methylation pattern of a CpG island located approximately 300 bp upstream of the miR-200c sequence in several cell lines. None of the cytosines in the analyzed CpG island were methylated in MCF-7 cells (Fig. 3a). By contrast, all the analyzed CpG sites were methylated in A549 cells and the level of DNA methylation was 60% in NCI-H460 cells. We next examined the expression of miR-200c in the same cell lines using quantitative RT-PCR. We found that the levels of miR-200c in MCF-7 cells were much higher than those in A549 and NCI-H460 cells (Fig. 3b). These results indicate a correlation between DNA hypomethylation and expression of miR-200c.

### Inhibition of miR-200c upregulates HIF-1α

Next, we examined whether the high levels of miR-200c in MCF-7 cells repress the expression of HIF-1α. For this purpose, MCF-7 cells were transfected with miR-200c inhibitor and cultured in normoxia or hypoxia. After 24 h, total proteins were extracted and subjected to western blot analysis. Transfection of miR-200c inhibitor in hypoxia resulted in an increase in the HIF-1α protein level, indicating that high levels of miR-200c negatively affect the expression of HIF-1α in MCF-7 cells under hypoxic conditions (Fig. 3c).

### miR-200c inhibits the migration of A549 cells

**Fig. 4:**
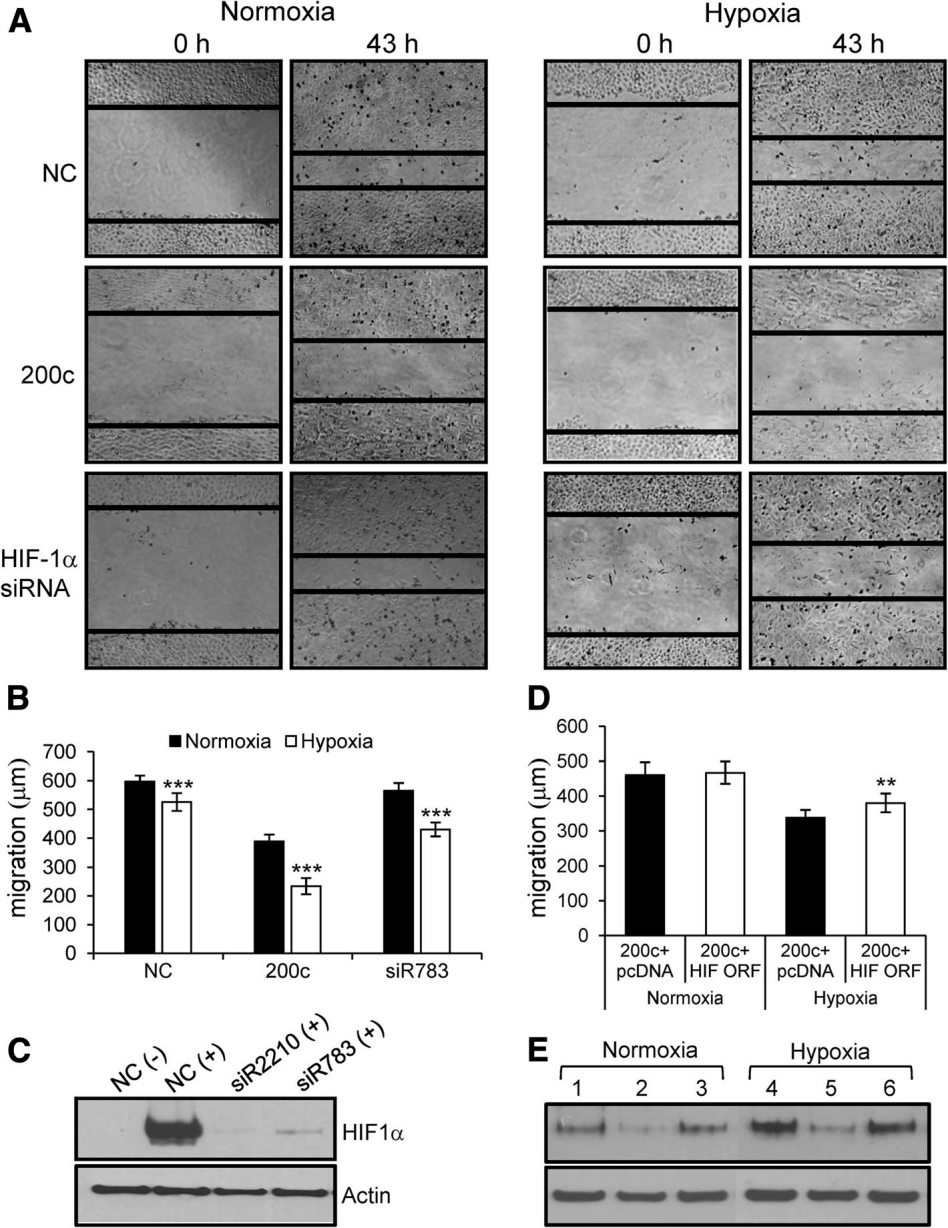
Wound-healing assays in A549 cells transfected with miR-200c mimic and siRNA against *HIF-1α.* **a** and **b –** In these assays, we used A549 cells because the cell migration of NCI-H460 cells was very slow under our experimental conditions. A549 cells were transfected with miR-200c mimic and siR783 (20 nM). After a scratch was made, the cells were grown for 43 h in normoxia or hypoxia. Images were taken 0 and 43 h after wound formation (**a**) and cell migration toward the wound region was measured (**b**). ***p < 0.001. **c –** Efficient knockdown of HIF-1α by siR783, as revealed via western blot analysis. In the wound-healing assays, we used siR783 as an siRNA that targets HIF-1α because siR2210 inhibited cell migration even in normoxia, most likely due to off-target effect. (−): no treatment with CoCl_2_; (+): treatment with CoCl_2._ **d** and **e –** Rescue experiments. A549 cells were first transfected with NC or miR-200c and 6 h later, transfected with pcDNA3.1 or pcDNA3.1-HIF-1α. After 43 h in normoxia or hypoxia, cells underwent cell migration evaluation (**d**) and western blot analysis (**e**). In (**e**), the notations are 1, 4: co-transfected with NC and pcDNA3.1; 2, 5: co-transfected with miR-200c and pcDNA3.1; 3, 6: co-transfected with miR-200c and pcDNA3.1-HIF-1α. For western blot analyses, the pcDNA3.1 and pcDNA3.1-HIF-1α plasmids were transfected at 70 ng/2 ml in a 6-well plate. **p < 0.01

To examine whether miR-200c affects cell migration, we performed wound-healing assays. The results showed that miR-200c inhibits cell migration in normoxia and to a greater extent in hypoxia (Fig. 4a and b). Since HIF-1α silencing inhibits cell migration in hypoxia but not in normoxia, downregulation of HIF-1α by miR-200c appears to contribute to a greater inhibition of cell migration by miR-200c in hypoxic conditions.

To examine this possibility, we performed rescue experiments using pcDNA3.1-HIF-1α plasmid expressing HIF-1α. Co-transfection of miR-200c and pcDNA3.1-HIF-1α plasmid restored the level of HIF-1α and increased cell migration by 12.4% in hypoxia, but not in normoxia (Fig. 4d and e). This increase corresponds to 28.9% of the difference in cell migration between normoxia and hypoxia. These results indicate that downregulation of HIF-1α by miR-200c contributes to the inhibition of cell migration in hypoxia.

## Discussion

In this study, we found that HIF-1α is downregulated by miR-18, −200c and − 549 at the mRNA and protein levels in lung carcinoma cells. Luciferase assays revealed that *HIF-1α* was a direct target of miR-18 and miR-549. Intriguingly, we did not find any potential matching 6/7-mer seed sequence in miR-200c and the 3′-UTR of *HIF-1α.* As expected, the TargetScan program did not predict *HIF-1α* as a target of miR-200c. Recently, the Ago-HITS-CLIP (argonaute high-throughput sequencing after cross-linked immunoprecipitation) technology has been used to identify targets of miR-200a and miR-200b at the genome level. *HIF-1α* was one such target [22]. Another study similarly reported that miR-200b directly targets *HIF-1α* in colorectal cancer cells [23]. The authors showed that transfection with miR-200b inhibitor increased HIF-1α levels, identified a putative miR-200b target sequence in the 3′-UTR of *HIF-1α* using the RNA hybrid software, and confirmed the binding of miR-200b to this sequence in luciferase reporter assays. Notably, the target sequence that Shang et al. identified was not a canonical seed sequence.

We thought that a similar phenomenon could happen in the interaction between miR-200c and the 3′-UTR of *HIF-1α* in lung cancer cells. To validate the target sequence identified by Shang et al. as a functional binding site for miR-200c in lung cancer cells, we mutated it and performed luciferase reporter assays. Our result showed that the luciferase activity increased only slightly in cells overexpressing miR-200c when we transfected them with the mutant reporter plasmid (wild-type: 73.8%; mutant: 77.5%). Therefore, additional studies are necessary to identify how miR-200c exerts its effect on *HIF-1α* mRNA in lung cancer cells.

Increasing evidence indicates that members of the miR-200 family inhibit cell migration and invasion [24–26]. Recently, Bracken et al. identified hundreds of targets of miR-200a/b using the Ago-HITS-CLIP technology [22]. Gene ontology analysis revealed that the largest number of target genes of miR-200a/b are involved in cytoskeletal remodeling. Downregulation of multiple target genes involved in cytoskeletal remodeling resulted in cooperative inhibition of cell migration and invasion in breast cancer cells.

Our results also showed that overexpression of miR-200c in A549 lung carcinoma cells inhibited migration in wound-healing assays in both normoxia and hypoxia. In hypoxia, HIF-1α promotes cell migration and invasion by inducing multiple genes, including the stromal cell-derived factor-1/C-X-C motif chemokine receptor-4 (SDF-1/CXCR-4) axis, C-C motif chemokine receptor 7 (CCR7), and lysyl oxidase (LOX) in lung cancer cells [27–30]. Thus, downregulation of HIF-1α by miR-200c in hypoxia resulted in reduced migration compared with that in normoxia (Fig. 4b). In rescue experiments, restoration of HIF-1α expression in miR-200c-transfected cells resulted in an increase in cell migration in hypoxia but not in normoxia, indicating that miR-200c plays a role in the inhibition of cell migration by downregulating HIF-1α. The partial increase in the cell migration of HIF-1α-expressing cells under hypoxic conditions imply that additional targets may be involved in the inhibition of cell migration by miR-200c in lung cancer cells.

To date, few studies have examined the role of the miR-200 family in cellular processes in hypoxia. LOX was previously found to be a direct target of miR-200b and a downstream target of HIF-1α. Thus, LOX is downregulated by miR-200b directly and indirectly by targeting of HIF-1α, which in turn reduces hypoxic induction of the LOX gene. RNAi experiments revealed that downregulation of LOX after siRNA treatment inhibited hypoxia-induced migration and invasion in non-small cell lung cancer [30, 31]. Another example is the transcription factor Ets-1, which is also directly targeted by miR-200b. Interestingly, Ets-1 plays a role in selecting HIF-2α target genes [32]. Knockdown of ELK-1, a member of the Est family, resulted in the reduction of hypoxic induction of HIF-2α-dependent genes. Thus, targeting of Est-1 by miR-200b may affect cellular processes, including cell migration under hypoxia, independently of HIF-1α.

In our preliminary experiments using microarray analyses, 79 genes involved in cell migration were upregulated more than 1.5-fold in A549 cells under hypoxia compared to normoxia. Of these, 11 genes were putative target genes of miR-200c as predicted by TargetScan. With the exception of VEGFA, the roles of 10 genes in cell migration under hypoxia in lung cancer cells have not been characterized. Additional studies are necessary to determine whether 1) these genes are direct targets of miR-200c; 2) their transcriptional induction under hypoxia depends on HIF-1α; and 3) they play a role in cell migration under hypoxia in lung cancer cells.

In addition to its established roles in EMT and angiogenesis, the effect of miR-200c on HIF-1α strengthens its tumor suppressive activity and therapeutic potential for cancer treatment through the inhibition of hypoxia-induced cellular processes.

## Conclusion

We found that miR-200c downregulates HIF-1α, thereby adversely affecting the cellular responses to hypoxia. Our results expand the role of miR-200c beyond its effect on EMT and angiogenesis to the regulation of the hypoxic response. Because of its role as a tumor suppressor, the introduction of miR-200c mimics into tumors and endothelial cells may have a therapeutic value for cancer treatment, by suppressing tumor metastasis and hypoxic responses, including angiogenesis.

## Additional file

Additional file 1: **Table S1.** Primers used for quantitative RT-PCR. (DOCX 21 kb)

## Abbreviations

3′-UTR: 3′-Untranslated region
Ago-HITS-CLIP: Argonaute high-throughput sequencing after cross-linked immunoprecipitation
ALDOA: Fructose-bisphosphate aldolase
CA9: Carbonic anhydrase 9
CCR7: C-C motif chemokine receptor 7
CoCl_2_: Cobalt chloride
CXCR4: C-X-C motif chemokine receptor 4
EMT: Epithelial–mesenchymal transition
Ets-1: V-ets erythroblastosis virus E26 oncogene homolog 1
Flt-1: *fms*-like tyrosine kinase
HIF-1α: hypoxia-inducible factor-1α
HRE: Hypoxia-responsive element
KDR: Kinase-insert domain-containing receptor
LDHA: Lactate dehydrogenase A
LOX: Lysyl oxidase
miRNA: MicroRNA
NC: Negative control
ORF: Open reading frame
PGK1: Phosphoglycerate kinase 1
PHD: Prolyl hydroxylase domain
PKM: Pyruvate kinase muscle
SDF-1: Stromal cell-derived factor 1
siRNAs: Short interfering RNAs
VEGF: Vascular endothelial growth factor
VHL: Von Hippel-Lindau
ZEB: Zinc finger E-box-binding homeobox

## References

[1] Ke Q, Costa M (2006). Hypoxia-inducible factor-1 (HIF-1). Mol Pharmacol.

[2] Jaakkola P, Mole DR, Tian YM, Wilson MI, Gielbert J, Gaskell SJ (2001). Targeting of HIF-alpha to the von Hippel-Lindau ubiquitylation complex by O2-regulated prolyl hydroxylation. Science..

[3] Masson N, Willam C, Maxwell PH, Pugh CW, Ratcliffe PJ (2001). Independent function of two destruction domains in hypoxia-inducible factor-alpha chains activated by prolyl hydroxylation. EMBO J.

[4] Maxwell PH, Wiesener MS, Chang GW, Clifford SC, Vaux EC, Cockman ME (1999). The tumour suppressor protein VHL targets hypoxia-inducible factors for oxygen-dependent proteolysis. Nature..

[5] Liu W, Shen SM, Zhao XY, Chen GQ (2012). Targeted genes and interacting proteins of hypoxia inducible factor-1. Int J Biochem Mol Biol.

[6] Hu Y, Liu J, Huang H (2013). Recent agents targeting HIF-1α for cancer therapy. J Cell Biochem.

[7] Gregory PA, Bert AG, Paterson EL, Barry SC, Tsykin A, Farshid G (2008). The miR-200 family and miR-205 regulate epithelial to mesenchymal transition by targeting ZEB1 and SIP1. Nat Cell Biol.

[8] Park SM, Gaur AB, Lengyel E, Peter ME (2008). The miR-200 family determines the epithelial phenotype of cancer cells by targeting the E-cadherin repressors ZEB1 and ZEB2. Genes Dev.

[9] Chan YC, Khanna S, Roy S, Sen CK (2011). miR-200b targets Ets-1 and is down-regulated by hypoxia to induce angiogenic response of endothelial cells. J Biol Chem.

[10] Roybal JD, Zang Y, Ahn YH, Yang Y, Gibbons DL, Baird BN (2011). miR-200 inhibits lung adenocarcinoma cell invasion and metastasis by targeting Flt1/VEGFR1. Mol Cancer Res.

[11] Choi YC, Yoon S, Jeong Y, Yoon J, Baek K (2011). Regulation of vascular endothelial growth factor signaling by miR-200b. Mol Cells.

[12] Choi YC, Yoon S, Byun Y, Lee G, Kee H, Jeong Y (2015). MicroRNA library screening identifies growth-suppressive microRNAs that regulate genes involved in cell cycle progression and apoptosis. Exp Cell Res.

[13] Kim S, Lee UJ, Kim MN, Lee EJ, Kim JY, Lee MY (2008). MicroRNA miR-199a* regulates the MET proto-oncogene and the downstream extracellular signal-regulated kinase 2 (ERK2). J Biol Chem.

[14] Livak KJ, Schmittgen TD (2001). Analysis of relative gene expression data using real-time quantitative PCR and the 2(−Delta Delta C(T)) method. Methods..

[15] Piret JP, Mottet D, Raes M, Michiels C (2002). CoCl2, a chemical inducer of hypoxia-inducible factor-1, and hypoxia reduce apoptotic cell death in hepatoma cell line HepG2. Ann N Y Acad Sci.

[16] Wu F, Huang W, Wang X (2015). microRNA-18a regulates gastric carcinoma cell apoptosis and invasion by suppressing hypoxia-inducible factor-1α expression. Exp Ther Med.

[17] Han F, Wu Y, Jiang W (2015). MicroRNA-18a decreases choroidal endothelial cell proliferation and migration by inhibiting HIF1A expression. Med Sci Monit.

[18] Grabmaier K, A de Weijert MC, Verhaegh GW, Schalken JA, Oosterwijk E (2004). Strict regulation of CAIX(G250/MN) by HIF-1alpha in clear cell renal cell carcinoma. Oncogene..

[19] Raval RR, Lau KW, Tran MG, Sowter HM, Mandriota SJ, Li JL (2005). Contrasting properties of hypoxia-inducible factor 1 (HIF-1) and HIF-2 in von Hippel-Lindau-associated renal cell carcinoma. Mol Cell Biol.

[20] Jones PA, Takai D (2001). The role of DNA methylation in mammalian epigenetics. Science..

[21] Klose RJ, Bird AP (2006). Genomic DNA methylation: the mark and its mediators. Trends Biochem Sci.

[22] Bracken CP, Li X, Wright JA, Lawrence DM, Pillman KA, Salmanidis M (2014). Genome-wide identification of miR-200 targets reveals a regulatory network controlling cell invasion. EMBO J.

[23] Shang Y, Chen H, Ye J, Wei X, Liu S, Wang R (2017). HIF-1α/Ascl2/miR-200b regulatory feedback circuit modulated the epithelial-mesenchymal transition (EMT) in colorectal cancer cells. Exp Cell Res.

[24] Howe EN, Cochrane DR, Richer JK (2011). Targets of miR-200c mediate suppression of cell motility and anoikis resistance. Breast Cancer Res.

[25] Korpal M, Lee ES, Hu G, Kang Y (2008). The miR-200 family inhibits epithelial-mesenchymal transition and cancer cell migration by direct targeting of E-cadherin transcriptional repressors ZEB1 and ZEB2. J Biol Chem.

[26] Liu L, Qiu M, Tan G, Liang Z, Qin Y, Chen L (2014). miR-200c inhibits invasion, migration and proliferation of bladder cancer cells through down-regulation of BMI-1 and E2F3. J Transl Med.

[27] Liu YL, Yu JM, Song XR, Wang XW, Xing LG, Gao BB (2006). Regulation of the chemokine receptor CXCR4 and metastasis by hypoxia-inducible factor in non small cell lung cancer cell lines. Cancer Biol Ther.

[28] Li Y, Qiu X, Zhang S, Zhang Q, Wang E (2009). Hypoxia induced CCR7 expression via HIF-1alpha and HIF-2alpha correlates with migration and invasion in lung cancer cells. Cancer Biol Ther..

[29] Erler JT, Bennewith KL, Nicolau M, Dornhöfer N, Kong C, Le QT (2006). Lysyl oxidase is essential for hypoxia-induced metastasis. Nature..

[30] Wei L, Song XR, Sun JJ, Wang XW, Xie L, Lv LY (2012). Lysyl oxidase may play a critical role in hypoxia-induced NSCLC cells invasion and migration. Cancer Biother Radiopharm.

[31] Sun M, Gomes S, Chen P, Frankenberger CA, Sankarasharma D, Chung CH (2014). RKIP and HMGA2 regulate breast tumor survival and metastasis through lysyl oxidase and syndecan-2. Oncogene..

[32] Aprelikova O, Wood M, Tackett S, Chandramouli GV, Barrett JC (2006). Role of ETS transcription factors in the hypoxia-inducible factor-2 target gene selection. Cancer Res.

