# Supplementary material for: MiR-200c downregulates HIF-1α and inhibits migration of lung cancer cells
Source: Cell Mol Biol Lett. 2019 Apr 27;24:28. doi: 10.1186/s11658-019-0152-2 (PMC6487019; doi:10.1186/s11658-019-0152-2)
Supplement: Supplementary file 1 — Table S1. Primers used for quantitative RT-PCR. (DOCX 21 kb) [file 11658_2019_152_MOESM1_ESM.docx]

| Name | Sequence (5′ - 3′) |
| --- | --- |
| β-Actin | Forward: TCCCTGGAGAAGAGCTACGA  Reverse: AGGAAGGAAGGCTGGAAGAG |
| ALDOA | Forward: GCGTTGTGTGCTGAAGATTG  Reverse: GCTGGCAGATACTGGCATAA |
| CA9 | Forward: TCAGCCGCTACTTCCAATATG  Reverse: TCAGCATCACTGTCTGGTTAAA |
| HIF-1α | Forward: CCCATTCCTCACCCATCAAATA  Reverse: CTTCTGGCTCATATCCCATCAA |
| LDHA | Forward: GAAGACTCTGCACCCAGATTTA  Reverse: TCACCTCATAAGCACTCTCAAC |
| PGK1 | Forward: TGCATCTCCACTTGGCATTAG  Reverse: GATGCTGTGCAACTGTTTAAGG |
| PKM | Forward: TCTGGAGAAACAGCCAAAGG  Reverse: CGGCGGAGTTCCTCAAATAA |

Table S1. Primers used for quantitative RT-PCR
